# Contrast Sensitivity versus Visual Evoked Potentials in Multiple Sclerosis

**Published:** 2010-07

**Authors:** Javad Heravian Shandiz, Abbas Nourian, Mercedeh Bahr Hossaini, Hadi Ostadi Moghaddam, Abbas-Ali yekta, Laleh Sharifzadeh, Parviz Marouzi

**Affiliations:** 1 Department of Optometry, Paramedical Faculty, Mashhad University of Medical Sciences, Mashhad, Iran; 2 Arya Hospital, Islamic Azad Medical University, Mashhad, Iran; 3 Department of Epidemiology, Health Faculty, Mashhad University of Medical Sciences, Mashhad, Iran

**Keywords:** Visual Evoked Potential, Contrast Sensitivity, Multiple Sclerosis

## Abstract

**Purpose:**

To compare the Cambridge contrast sensitivity (CS) test and visual evoked potentials (VEP) in detecting visual impairment in a population of visually symptomatic and asymptomatic patients affected by clinically definite multiple sclerosis (MS).

**Methods:**

Fifty patients (100 eyes) presenting with MS and 25 healthy subjects (50 eyes) with normal corrected visual acuity were included in this study. CS was determined using the Cambridge Low Contrast Grating test and VEP was obtained in all eyes. Findings were evaluated in two age strata of 10–29 and 30–49 years.

**Results:**

Of the 42 eyes in the 10–29 year age group, CS was abnormal in 22 (52%), VEP was also abnormal in 22 (52%), but only 12 eyes (28%) had visual symptoms. Of the 58 eyes in the 30–49 year group, CS was abnormal in 7 (12%), VEP was abnormal in 34 (58%), while only 11 eyes were symptomatic. No single test could detect all of the abnormal eyes.

**Conclusion:**

The Cambridge Low Contrast Grating test is useful for detection of clinical and subclinical visual dysfunction especially in young patients with multiple sclerosis. Nevertheless, only a combination of CS and VEP tests can detect most cases of visual dysfunction associated with MS.

## INTRODUCTION

Patients with multiple sclerosis (MS) often demonstrate visual dysfunction which may be clinical or subclinical. The detection of occult visual loss is important in establishing a diagnosis of multiple sclerosis, especially in patients who have neurological symptoms of the disease. Snellen acuity loss, color deficiencies, static perimetric defects, and optic atrophy are characteristic findings following optic neuritis.^1^ In many patients with multiple sclerosis, conventional visual tests (i.e., visual acuity and visual fields) may not detect all functional visual deficits. In some MS patients visual acuity may be normal even when patients complain of poor vision which they often describe as “blurred” or “washed out”.^2^ Subtle visual abnormalities may be revealed by more specific examinations.^1^ Alteration in visual evoked potential (VEP) latencies using pattern stimuli is considered one of the most characteristic electrophysiological signs observed in patients with MS. Additionally, subjective psychophysical tests such as contrast sensitivity (CS) have been reported to be useful in detection of visual abnormalities, but have been judged to be more or less sensitive than other methods according to different studies.^3^

Contrast sensitivity is defined as the threshold contrast at which a faint pattern of stripes is just visible. Impairment of CS has been demonstrated in a variety of disorders associated with visual loss. CS testing unmasks “hidden visual loss” in multiple sclerosis and has been proposed as a simple and rapid screening test for subclinical optic neuropathy.^4^

In this study we compare the Cambridge CS test and VEP for detection of visual dysfunction in a population of visually symptomatic and asymptomatic patients with clinically definite MS.

## METHODS

Fifty patients (100 eyes) diagnosed with clinically definite multiple sclerosis and normal or corrected-to-normal Snellen acuity (6/6) were selected from a neurology clinic; 39 patients were female, 11 were male and mean age was 30.8±5.7 (range, 17–45) years.

Normative data for CS and VEP were derived from 25 age-matched subjects with normal Snellen acuity who were selected from students and patients with non-neurological disease. These control subjects included 19 female and 6 male individuals with mean age of 29.5±5.6 (range, 19–41) years. All control subjects and patients underwent VEP and CS testing. None of the controls had ocular abnormalities affecting contrast sensitivity. Furthermore, all eyes had Snellen acuity of 6/6 or more, after full optical correction.

Contrast sensitivity was assessed using the Cambridge Low Contrast Grating test^5^ (Clement Clarke, London, UK) at a viewing distance of 6 meters with full correction, if necessary. Each eye was tested separately. This test includes 12 pairs of plates. The luminance of the test plates is about 100cd/m^2^. Series of plate pairs were presented in order of increasing difficulty to a total of four times for each eye. The observer was told to choose whether the top or bottom plate contained the grating, guessing if necessary. Using a conversion table, the maximum possible score was recorded for each eye.

VEP was recorded using the Toennies Neuroscreen (Jaeger-Toennies Inc., Höchberg, Germany). VEPs were elicited by checkerboards reversing at 1 Hz (2 reversals per second) on a television monitor located 1m from the patient. The VEP program provides a checkerboard pattern sized 15 minutes of arc for visual stimulation^6^. Checkerboard contrast was 80% with luminance of 89 cd/m^2^. VEP was recorded by means of bipolar midline derivation with the active electrode located 2.5 cm above the inion referenced to the center of the forehead with a ground electrode attached to the right wrist by a clip. The amplifier bandwidth filters were set at 1.0–100 Hz. VEP waves, consisting of 300 sweeps, were randomly recorded for each eye. Averages were plotted on an x-plotter and measured latencies were compared with norms. VEP measurements were obtained with the patient wearing a visual aid, if needed and the fellow eye was occluded. A fixation spot was used on the center of the screen during stimulation. The latency of the first major positive peak in the VEP (P_100_ wave) was measured. Most investigators currently interpret pattern reversal VEP (P-VEP) based almost entirely on latency of P_100_.

Whenever applicable, paired sample and independent t-tests were performed to compare means. P-values less than 0.05 were considered as significant. Cohen’s Kappa coefficient was used to measure inter-rater agreement among parameters in eyes with abnormal CS, delayed VEP and visual symptoms.

## RESULTS

Patients and control subjects were divided into two age strata of 10–29 and 30–49 years. Of 50 patients (100 eyes) with clinically definite MS, 21 subjects (42 eyes) were aged 10–29 years and 29 others (58 eyes) were 30–49 years of age. CS and VEP scores of control subjects were recorded in order to determine the normal limits (2 standard deviations from the mean value) within each group. Mean and standard deviations for CS and P_100_ latency in both age groups in controls and patients are shown in Table 1. Contrast sensitivity scores in all control subjects but one (96%) were within normal range. All control subjects (100%) had normal P_100_ latencies. It should be noted that the normal VEP latency in this study was less than 114 msec.

Of the 42 eyes in the 10–29 year age group, CS was abnormal in 22 eyes (52%), VEP was also abnormal in 22 eyes (52%), but only 12 eyes (29%) had visual symptoms. Ten out of 42 eyes (24%) had both abnormal CS and VEP, 12 eyes (29%) only had an abnormal CS and 12 others (29%) only had an abnormal VEP. Eight eyes (18%) demonstrated no abnormality on either test.

Of 58 eyes in the 30–49 year age group, CS was abnormal in 7 eyes (12%), whereas VEP was abnormal in 34 eyes (59%), but only 11 eyes (19%) were symptomatic. Four out of 58 eyes (7%) had both an abnormal CS and VEP, 3 eyes (5%) only had an abnormal CS and 30 others (52%) only had an abnormal VEP. Twenty-one (36%) eyes demonstrated no abnormality on either test.

The association between abnormal VEP and CS tests was not statistically significant in any of the study groups. There was a statistically significant decrease in contrast sensitivity with age (P<0.001), whereas no significant trend was observed for VEP latency or visual symptoms as a function of age.

**Figure 1:**
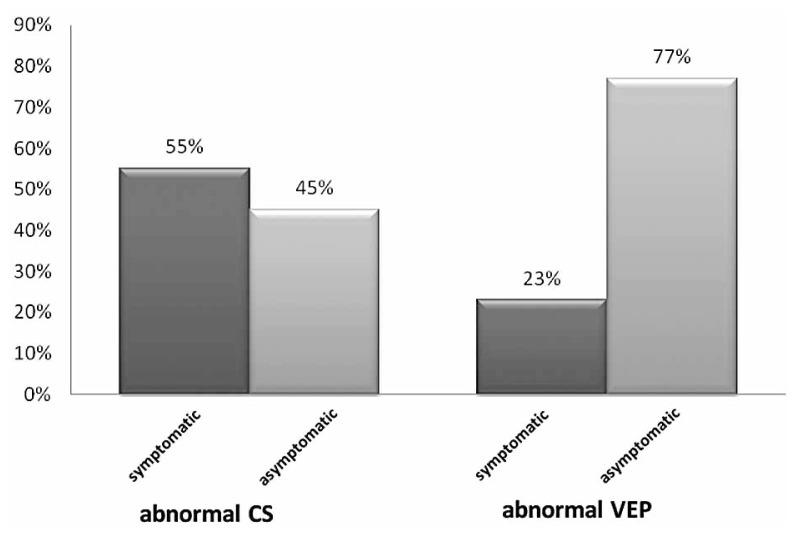
The frequency of symptomatic and asymptomatic eyes in patients with abnormal CS and VEP in the 10–29 year age group.

In the young age group, among 22 eyes with abnormal CS, 12 (55%) were visually symptomatic and 10 (45%) were asymptomatic. In the same group, of 22 eyes with abnormal VEP, only 5 (23%) had visual symptoms and 17 (77%) were asymptomatic (Fig. 1).

**Figure 2:**
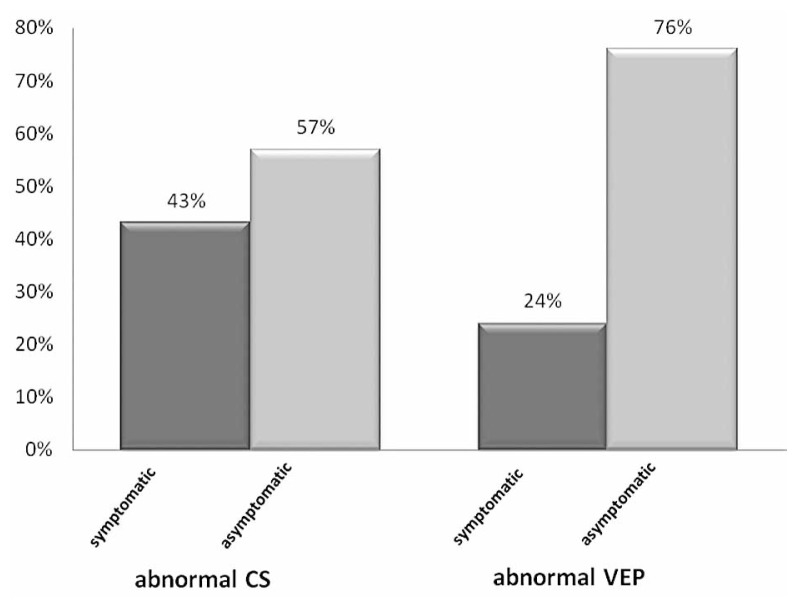
The frequency of symptomatic and asymptomatic eyes in patients with abnormal CS and VEP in the 30–49 year age group.

In the older age group, among 7 eyes with abnormal CS, 3 (43%) were symptomatic and 4 (57%) were asymptomatic. In the same group, among 34 eyes with abnormal VEP, only 8 (24%) had visual symptoms and 26 (76%) were asymptomatic (Fig. 2).

The correlation between abnormal CS and visual symptoms such as blurred vision was significant only in the 10–29 year age group (kappa=0.533, P=0.001). Visual symptoms and delayed VEP failed to show any significant correlation in either of the age strata.

The prevalence of abnormalities in monocular and binocular contrast sensitivity was the same in the 10–29 year age group (13 out of 21 patients, 62%). In the 30–49 year age group, only 5 out of 29 patients (17%) had abnormal monocular CS, whereas abnormal binocular CS was observed in 13 of the 29 patients (45%) in this age group. The comparison between abnormal monocular and binocular CS indicated a significant difference in this age group (P<0.001). No significant decrease in binocular contrast sensitivity was noted with age.

**Figure 3:**
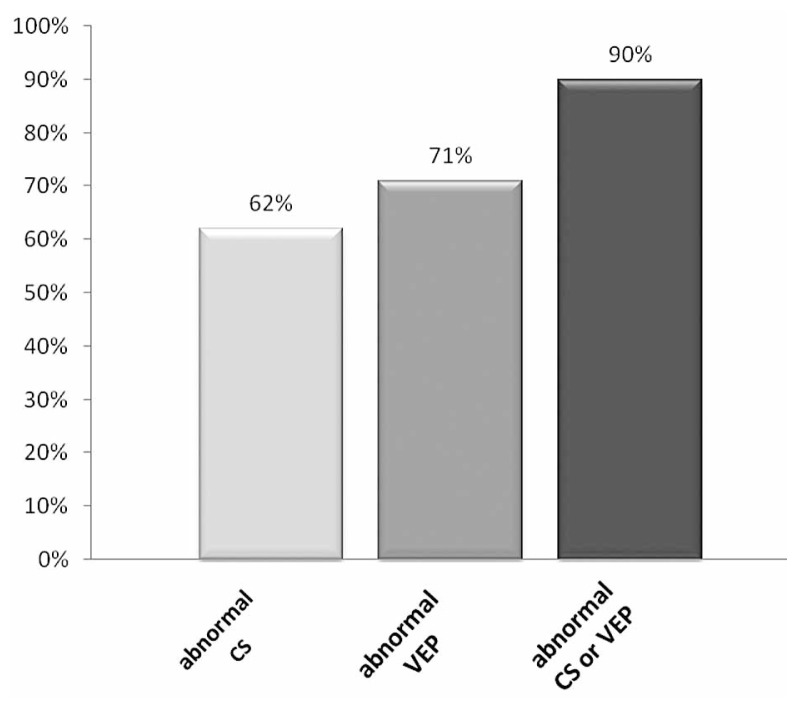
The frequency of patients with abnormal CS, abnormal VEP and abnormal CS or VEP in the 10–29 year age group.

**Figure 4:**
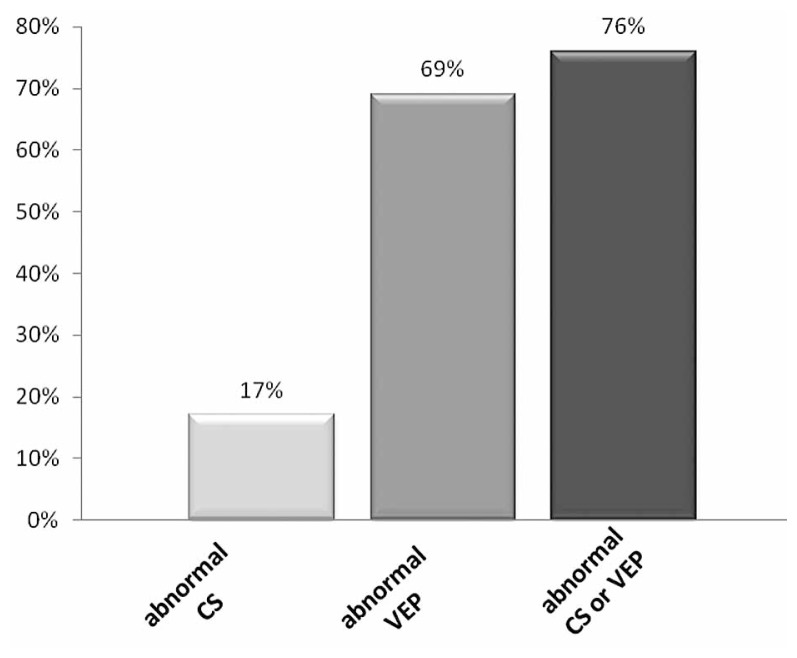
The frequency of patients with abnormal CS, abnormal VEP and abnormal CS or VEP in the 30–49 year age group.

Considering both eyes of each patient together, of 21 patients in the young age group, CS was abnormal in 13 (62%) and VEP was abnormal in 15 (71%) subjects in one or both eyes. Of 29 patients in the older age group, CS was abnormal in 5 (17%) and VEP was delayed in 20 (69%) subjects in one or both eyes. When both tests were taken together, 90% of patients in the younger age group and 76% of those in the older age group had either an abnormal CS or VEP (Figures 3, 4).

## DISCUSSION

This study revealed that CS and VEP both detected the same rate of abnormalities in young patients with clinically definite MS. In contrast, VEP showed a far higher rate of abnormalities than CS in older subjects. Many other studies have shown the presence of subclinical alterations of the visual system in MS, but there is little agreement on the prevalence of these abnormalities or the most sensitive diagnostic tests.

Fahy et al^4^ compared CS impairment and VEP abnormalities among 39 patients with clinically definite multiple sclerosis and reported that CS was impaired in only 33% of patients while VEP was abnormal in 82%. Della Sala et al^7^ studied 48 multiple sclerosis patients using the same tests and found that 73% had abnormal CS and 82% had delayed VEP. Van Diemen et al^8^ evaluated the visual system in 22 patients with clinically definite multiple sclerosis without visual symptoms. They found CS abnormalities in 72% and VEP abnormalities in 81%. Sisto et al^3^ evaluated subclinical visual involvement in multiple sclerosis among 11 patients and reported abnormal CS in 17 (77.1%) and delayed VEP in 12 eyes (54.4%). Leys et al^9^ found an abnormal CS in only 50% and abnormal VEP in 94% of a total of 18 multiple sclerosis patients. Wender^10^ studied contrast sensitivity in 100 patients with multiple sclerosis and found 59% had an abnormal CS. The large discrepancies in reported rates of CS and VEP abnormalities may be due to differences in examination methods and enrollment criteria.

According to our results, contrast sensitivity declines with age which is consistent with findings of previous studies. Ross et al^11^ studied the effect of age on contrast sensitivity among 70 subjects and reported that as with a number of other visual functions, contrast sensitivity declines with age, particularly in medium and high spatial frequencies. Fahy et al^4^ also mentioned CS testing to be less productive over the age of 40. The possible mechanism suggested by Elliott et al^12^ was neural loss within visual pathways with increasing age. Morrison and McGrath^13^ concluded that age-related deterioration is primarily caused by changes within the central nervous system rather than the optical media, the transmission quality of which remains unaffected. Whitaker and Elliott^14^ also suggested that under normal conditions, neural factors primarily account for the deterioration in visual quality experienced by older subjects. However, it is still uncertain whether such changes are caused by retinal and neural cell loss and degeneration, or occur secondary to reduced retinal illumination due to senile pupillary miosis and increased lens absorption by the greater light scatter of the aged eye^15^. In young subjects, whose eyes have not yet shown the effects of aging, deficits in contrast sensitivity resulting from retrobulbar pathology may be more apparent than the elderly, in whom ocular factors play a relatively important role^7^.

Our findings demonstrated that binocular contrast sensitivity testing provides a better measure of MS-related visual dysfunction than the monocular test over the age of 30 years. However, binocular and monocular CS detected the same rate of abnormalities in subjects less than 30. According to our results, as monocular contrast threshold increases by age, binocular summation appears to become more appreciable in older subjects. In contrast, Owsley et al^16^ demonstrated that younger observers manifest binocular summation effects greater than older observers. Pardhan^17^ compared binocular and monocular contrast sensitivity in young and elderly observers for sinusoidal gratings of 1 and 6 cycle/degree. This study also found lower binocular summation effects at both spatial frequencies for older subjects. It is noteworthy that in both studies mentioned above, healthy observers with different age ranges were assessed and different contrast sensitivity tests were employed.

We found no significant change in VEP abnormalities as a function of age, which supports previous studies. According to a study by Mitchell and Neville^18^, the visual pathway shows little age related change from young adulthood to middle ages (20–60 years). Della Sala et al^7^ also found no marked change in VEP abnormalities with age. In contrast, Tubimatsu et al^19^ showed the presence of a curvilinear relationship between P_100_ latency and age. The extent of the effect of age on VEP latency depends on stimulus parameters (luminance, contrast, and spatial frequency).^20^ The increase in P_100_ latency with age is more apparent at lower levels of luminance and contrast. Moreover, the age-related latency effect is greater for high (small checks) than for low (large checks) spatial frequencies, therefore these factors may affect age-related changes in VEP.^21^ The visual system consists of multiple, parallel channels which process information and aging may differentially affect specific functional subdivisions in the visual pathways.^21^ Furthermore, some have concluded that the increase in VEP latency with aging occurs mainly in older age strata (after the fifth decade)^22^ and most of the patients in our study, even in the older age group, were younger than 45. According to our results, VEP is more likely to detect optic neuropathy in the older age group.

A significant association was found between abnormal contrast sensitivity and visual symptoms only in the young age group of 10–29. This was probably due to the fact that CS was abnormal in all symptomatic eyes in this age group. We found that CS was more sensitive in detecting clinical deficits in both age groups. In contrast, VEP detected more subtle subclinical deficits. Leys et al^9^ also concluded that VEP was more sensitive than CS at detecting occult visual loss in patients with multiple sclerosis.

Despite the fact that neither VEP nor CS could detect all cases of MS, when the two tests were taken together, 90% of patients in the young age group and 75% of the older age group had at least one abnormal test result. These findings support those of previous authors. Van Diemen et al^8^ reported that the combination of the two tests detected abnormalities in 90.9% of their subjects. In the Della Sala et al^7^ study, 95% of abnormalities were found after the two tests were performed together and thus, they considered CS testing as a useful supplementary test for the evaluation of patients with suspected multiple sclerosis. Burki^22^ studied VEP and CS in 49 patients with optic neuritis and 26 multiple sclerosis patients with no previous history of optic neuritis and concluded that combined testing with CS and VEP was superior to a single test and detected 100% of acute optic nerve lesions.

According to our results, there was no significant correlation between VEP abnormalities and CS impairment, which is consistent with findings reported in previous studies.^7^,^8^ One possible explanation is that these tests measure different aspects of visual function. Therefore, the combination of the two tests can improve the detection of visual dysfunction in both symptomatic and asymptomatic eyes.

The Cambridge Low Contrast Grating test is inexpensive, portable, easy to score, and can examine contrast sensitivity in any meridian. It has the potential disadvantage of measuring contrast sensitivity at only one spatial frequency. On rare occasions, sensitivity loss can be more marked in certain spatial frequencies and any test that examines only one spatial frequency may therefore, in principle be less sensitive than those that examine the entire frequency spectrum^5^. This may explain the lower rate of abnormal contrast sensitivity in our report, in comparison to previous studies.

We may conclude that the Cambridge Low Contrast Grating test is a useful test to detect clinical and subclinical visual dysfunction, especially in young patients with multiple sclerosis. Nevertheless, the combination of CS and VEP enables detection of most cases of visual dysfunction in patients with clinically definite MS.

## Figures and Tables

**Table 1:** Mean values and standard deviations of contrast sensitivity and P_100_ latency in normal controls and multiple sclerosis patients stratified by age

		Contrast Senstivity			P_100_ Latency (msec)		
	
Age groups		Controls (Mean±SD)	Patients (Mean±SD)	t-test	Controls (Mean±SD)	Patients (Mean±SD)	t-test
RE	10–29	456±89	286±143	P<0.001	105±5	114±21	P=0.315
	30–49	320±67	293±139	P=0.551	104±3	124±22	P<0.001

LE	10–29	461±96	282±137	P<0.001	105±5	114±18	P=0.075
	30–49	339±103	301±123	P=0.368	104±3	121±21	P=0.015

Bin	10–29	531±48	327±123	P<0.001			
	30–49	476±75	357±132	P=0.006			

SD, standard deviation; RE, right eye; LE, left eye; Bin, binocular
